# Childhood correlates of adult TV viewing time: a 32-year follow-up of the 1970 British Cohort Study

**DOI:** 10.1136/jech-2014-204365

**Published:** 2014-08-21

**Authors:** L Smith, B Gardner, M Hamer

**Affiliations:** 1 Department of Epidemiology and Public Health, Health Behaviour Research Centre, University College London, London, UK; 2 Department of Epidemiology and Public Health, Physical Activity Research Group, University College London, London, UK

**Keywords:** PREVENTION, EPIDEMIOLOGY, PUBLIC HEALTH

## Abstract

**Background:**

To identify, using a longitudinal data set, parental and childhood correlates of adult television (TV) viewing time at 32-year follow-up.

**Method:**

Data were derived from the 1970 British Cohort Study, a longitudinal observational study of 17 248 British people born in a single week of 1970. The present analyses incorporated data from the age 10 and 42-year surveys. When participants were aged 10 years, their mothers provided information on how often participants watched TV and played sports (never/sometimes/often), and parents’ own occupation, as well as height and weight. A health visitor objectively assessed participants’ height and weight at age 10. Thirty-two years later, when participants were aged 42 years, they reported their daily TV viewing hours (none/0≤1/1<3/3<5/≥5), physical activity and health status. Associations between putative childhood and parental correlates and adult TV viewing time were investigated using logistic regression.

**Results:**

Valid data at both time points were available for 6188 participants. Logistic regression models showed that those who reported ‘often’ watching TV at baseline were significantly more likely to watch >3 h/days of TV at follow-up (OR 1.42, 95% CI 1.21 to 1.65), as were those whose father was from a lower socio-occupational class (intermediate, routine/manual) compared with managerial (OR 1.55, 95% CI 1.14 to 2.11; OR 2.05, 95% CI 1.47 to 2.87). Body mass index (BMI) at age 10 was inversely associated with high TV in adulthood (per unit increase; OR 0.93, 95% CI 0.90 to 0.96) although fathers BMI when the child was aged 10 was positively associated with high TV in adulthood (per unit increase; OR 1.04, 95% CI 1.02 to 1.06).

**Conclusions:**

Findings suggest that childhood TV viewing time tracks into adulthood. Parents’ health behaviours and social position appear to be associated with their children's viewing habits, which may have important implications for the direction of future policy and practice. Specifically, findings support the case for early life interventions, particularly on socioeconomic inequalities, as a way of preventing sedentary behaviour in later life.

## Introduction

A growing body of research shows prolonged sedentary behaviour—defined as any waking behaviour characterised by energy expenditure below 1.5 metabolic equivalents while in a sitting or reclined posture—is detrimental to adult health, even when adults are sufficiently active (eg, see, Edwardson *et al*^1^). Recent UK physical activity guidelines include recommendations to reduce sedentary activities (Start Active Stay Active). However, while correlates of physical activity have been well-researched,^2^ correlates and predictors of sedentary behaviours have received little attention. The few available studies of the correlates of sedentary behaviour have predominantly focused on children and adolescents.^3^ ^4^

One important component of leisure sedentary behaviour is television (TV) viewing. Krantz-Kent and Stewart reported that in 2007 US adults (aged 55–59 years) spent approximately 2.8 h a day viewing TV in comparison to 0.4 h a day reading and 0.6 h a day socialising and communicating.^5^ Although TV viewing need not be passive, in that viewers can perform physical activities while watching,^6^ it is thought that most TV viewing is sedentary and inactive.^7^ Moreover, TV viewing has been shown to correlate positively with other health risk behaviours, such as the consumption of energy dense foods and cigarette smoking.^8^ ^9^ A recent meta-analysis of prospective cohort studies that investigated relationships between TV viewing time and disease onset found that prolonged TV viewing was associated with increased risk of type 2 diabetes, cardiovascular disease and all-cause mortality.^10^ Interventions that reduce TV viewing, or seek to make TV viewing more active, may be beneficial for population health.

To successfully modify TV viewing patterns, correlates of the behaviour need to be identified. modifiable correlates (eg, physical environmental) provide possible targets/mechanisms via which behaviour change might be achieved, and non-modifiable (ie, demographic) correlates indicate which groups are most at risk and so most in need of intervention. A recent systematic review showed higher levels of TV viewing in adults to be associated with lower education, older age, unemployment or lower working hours, and higher body mass index (BMI), independent of sex.^11^ The review identified mixed evidence for associations between TV viewing and marital status, income and ethnicity. Higher TV viewing was also found to be associated with lower leisure time physical activity and having a TV in the bedroom. Over 76% of these studies were, however, of a cross-sectional nature, *which can* make interpretation of causality problematic. Moreover, no study investigated *childhood correlates* of adult TV viewing.

Physical activity and sedentary behaviour have been suggested to track from childhood to adulthood. Telama *et al*^12^ found that a high level of physical activity at age 9–18 significantly predicted a high level of adult activity. Biddle *et al*^13^ proposed that sedentary behaviours will track from childhood through adolescence to adulthood, though there is presently no empirical evidence available to test this relationship. If childhood sedentary behaviour does predict adulthood sedentary behaviour, successful interventions in childhood may have a long-term behavioural impact that is sustained into adulthood. Promoting healthy behaviours in childhood as opposed to intervening only in adulthood could therefore be an optimally effective strategy to prevent morbidity, as exposure to cardiometabolic risk factors throughout the life course may present the greatest health risks. Crucially, what is missing from the current literature is empirical evidence tracking TV viewing time from childhood through to adulthood and identified childhood and parental correlates of adult TV viewing time. The present analyses aims to (1) investigate if TV viewing time tracks from childhood through to adulthood, and (2) identify childhood correlates of adult TV viewing time, to contribute to the growing body of literature to inform policy and practice on the promotion of active lifestyles.

Adults’ occupation, BMI, and leisure time physical activity have been shown to be associated with adults’ sedentary behaviour.^11^ Moreover, using a more proximal outcome, previous research has found that parental adiposity and socioeconomic status are childhood predictors of adult obesity.^14^ We hypothesised that parental occupation (a strong indicator of early life socioeconomic position (SEP)) and BMI, as well as children's TV viewing time, participation in sport and BMI are predictors of adult TV viewing time.

## Method

The 1970 British Cohort Study (BCS70) follows the lives of 17 284 people born in England, Scotland and Wales in a single week of 1970.^15^ The present analyses incorporated data from the age 10 and age 42 surveys. At the age 10 survey, conducted in 1980/1981, parents provided informed consent and were interviewed about the child's home background, social experience, hospital admissions, accidents and a number of factors concerning the experiences of the child and the family. The information was gathered through a structured interview with the mother of the child, or if she was not available, with someone who had knowledge on the child's health and development. The age 42 survey was conducted in 2012/2013 and comprised of a 60-min face-to-face computer-assisted-personal-interview, which included a vocabulary task and a self-completion section. The present analysis focused only on the variables described below (hypothesised to be associated with TV viewing time in adulthood). Participants provided informed consent and all data collection on BCS70 has received full ethical approval. In accordance with the University College London Research Ethics Committee Guidance, ethical approval was not required to perform secondary analyses of anonymous health surveillance survey data.

### Variables at age 10

The cohort member's mother provided information regarding how often their child watched TV and played sports (categorised as: never/sometimes/often). The health visitor recorded height and body mass for the calculation of BMI. Parents provided information on their occupation, which was categorised using the 1970 and 1980 Office of Population Censuses and Surveys Classification of Occupations (Managerial/Professional/ Intermediate [skilled and non-skilled]/Routine and manual), and also provided self-reported weight and height, from which BMI was calculated.

### Variables at age 42

Respondents reported how many hours they spent watching TV per day (none/0≤1/1<3/3<5/≥5); frequency of participation in 15 types of physical activities and sports (every day/5–6 times a week/2–3 times a week/once a week/2–3 a month/less often/not in past 12 months); self-rated health (excellent/very good/good/fair/poor); and assessment of own weight (about right/underweight/overweight/very overweight).

### Statistical analysis

We examined associations between childhood exposures at aged 10 (TV viewing, sports, child's and father's BMI as continuous variables, father's occupational class,) and TV viewing at age 42 using logistic regression models. TV viewing at age 42 was categorised into a binary variable and higher TV viewing roughly reflected the upper third of the distribution (<3 h/days or ≥3 h/days). We calculated ORs and 95% CIs for the risk of high TV viewing at age 42. Initially we performed univariate analysis for each of the childhood exposures. We then mutually adjusted the models for each of the childhood exposures. Lastly, the models were adjusted for possible confounders in adulthood (sports participation at age 42, self-rated health and weight status). We wanted to examine whether results changed when we replaced fathers’ BMI with mothers BMI. In addition, previous research has consistently shown that SEP from birth has a cumulative effect on poor health in adulthood. Thus we wanted to examine if the association between fathers SEP and adult TV viewing was robust after adjustment for the participants own educational attainment. Therefore, we conducted several sensitivity analyses. First, mothers’ BMI was included instead of fathers’ BMI. Second, we added participants’ highest educational attainment at age 42 (none/GCSE or O-level/ A-level/higher education) as a covariate, since education is an indicator of SEP through adult life. All analyses were conducted using SPSS V.20.

## Results

**Figure 1:**
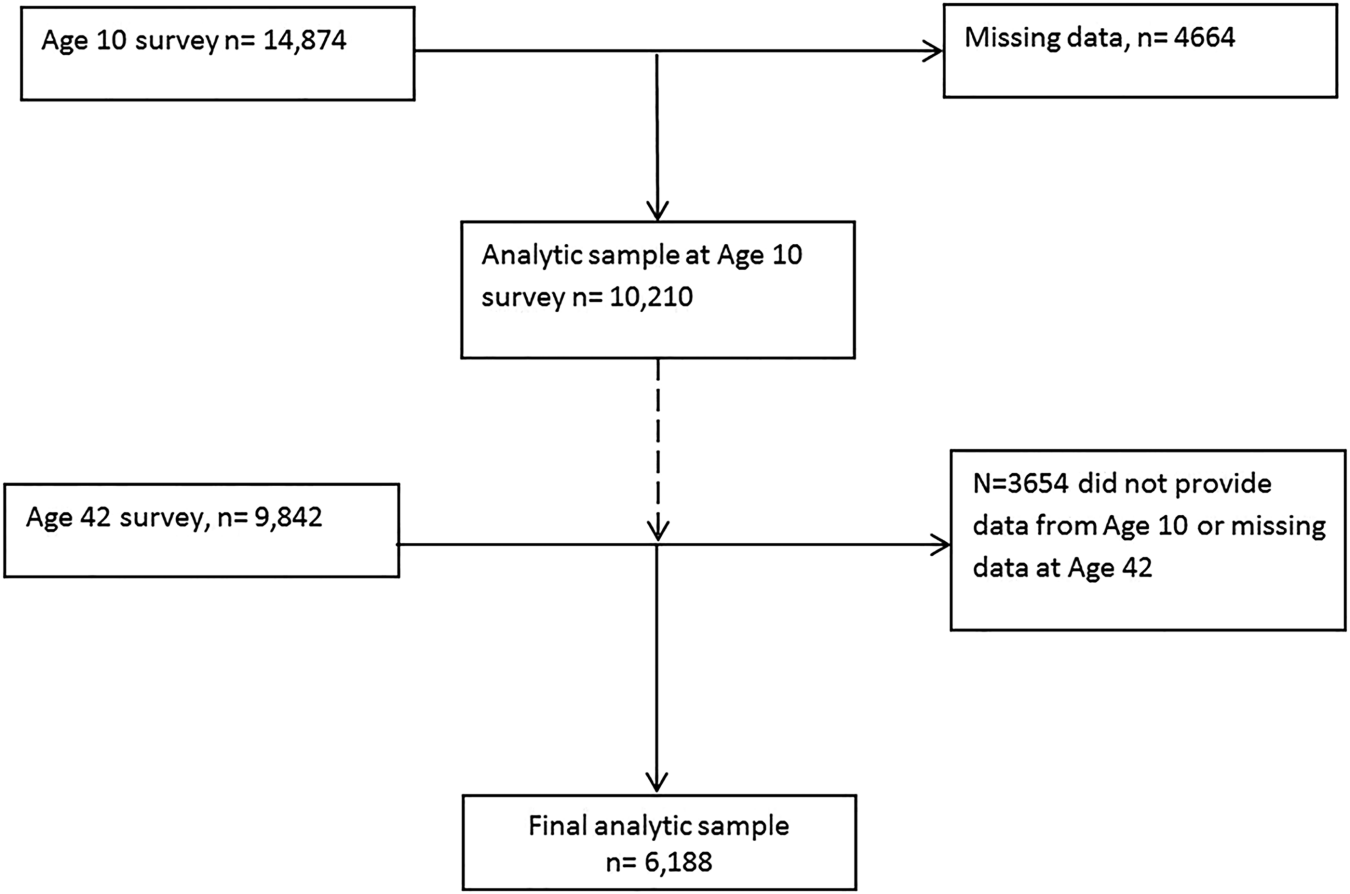
Flow diagram to present selection of analytic sample.

At the age 10 survey 14 874 cohort members participated, and 9842 (66.2%) took part in the age 42 survey. After excluding missing data from both assessments the final analytic sample comprised 6188 participants (41.6% of participants in age 10 survey; 62.9% of those in age 42 survey), which is described in figure 1. Compared with the analytic sample, those excluded from the final sample were more likely to be from a lower SEP background (% with father reporting routine/manual occupation: 14% vs 18.8%, p<0.001), more likely to report watching TV often at age 10 (78.5% vs 80.0%, p=0.031), and more likely to have a father with higher BMI (24.4 vs 24.6 kg/m^2^, p=0.007). No differences in sports participation were observed (53.8% vs 55.1%, p=0.17).

Of the 1546 participants who reported watching >3 h/days of TV at age 42 years, 50.2% were male, 82.9% often watched TV at age 10, 54.3% often played sport at age 10, 56.9% were from an intermediate socio-occupational class, 35.4% had a father in the upper third of the BMI distribution (>25.4 kg/m^2^; table 1).

**Table 1:** Descriptive characteristics of the sample at baseline (age 10) relative to TV viewing at follow-up (age 42)

Variable at baseline	Daily TV viewing at age 42			p Value
	<1 h/days (n=1061)	1–3 h/days (n=3581)	>3 h/days (n=1546)	
Gender				
Boys	456 (43.0)	1713 (47.8)	776 (50.2)	0.001
Girls	605 (57.0)	1868 (52.2)	770 (49.8)	
TV viewing at age 10				
Never/sometimes	285 (26.9)	787 (22.0)	265 (17.1)	<0.001
Often	776 (73.1)	2794 (78.0)	1281 (82.9)	
Playing sports at age 10				
Never/sometimes	523 (49.3)	1626 (45.4)	706 (45.7)	0.08
Often	538 (50.7)	1955 (54.6)	840 (54.3)	
Body mass index at age 10 (kg/m^2^)	16.9±2.0	16.9±2.1	16.8±2.1	0.20
Fathers body mass index (at child age 10 years)	24.1±2.9	24.3±2.9	24.7±3.1	0.001
Fathers’ socio-occupational class (child's age 10 years)				
Managerial	120 (11.3)	263 (7.3)	58 (3.8)	<0.001
Professional	370 (34.9)	995 (27.8)	306 (19.8)	
Intermediate (skilled and non-skilled)	454 (42.8)	1872 (52.3)	880 (56.9)	
Routine/manual	117 (11.0)	451 (12.6)	302 (19.5)	

Percentages denoted in brackets.

### Longitudinal associations between childhood characteristics and TV viewing at age 42

Final adjusted logistic regression models (table 2) showed that those who reported often watching TV at 10 years were significantly more likely to watch >3 h/days of TV at follow-up (OR 1.42, 95% CI 1.21 to 1.65), as were those whose father was from a lower socio-occupational class (intermediate, routine/manual) compared with managerial (OR 1.55, 95% CI 1.14 to 2.11; OR 2.05, 95% CI 1.47 to 2.87). BMI at age 10 was inversely associated with high TV in adulthood (per unit increase; OR 0.93, 95% CI 0.90 to 0.96, p-trend <0.001) although fathers BMI when the child was aged 10 was positively associated with high TV in adulthood (per unit increase; OR 1.04, 95% CI 1.02 to 1.06, p-trend=0.001).

**Table 2:** Association between baseline characteristics (child's age 10) and odds of high TV viewing (>3 h/days) at follow-up (age 42; n=6188)

Baseline independent variable	OR (95% CI) for high TV		
	Univariate model	Multivariate model 1*	Multivariate model 2†
Gender			
Boys	Ref	Ref	Ref
Girls	0.87 (0.78 to 0.98)	0.89 (0.78 to 0.99)	0.90 (0.78 to 1.03)
TV viewing at age 10			
Never/sometimes	Ref	Ref	Ref
Often	1.45 (1.29 to 1.69)	1.41 (1.21 to 1.64)	1.42 (1.21 to 1.65)
Playing sports at age 10			
Never/sometimes	Ref	Ref	Ref
Often	1.03 (0.91 to 1.15)	0.96 (0.85 to 1.08)	0.98 (0.87 to 1.11)
Childs body mass index at age 10 (per unit increase)	0.98 (0.95 to 1.00)	0.96 (0.93 to 0.98)	0.93 (0.90 to 0.96)
Fathers body mass index per unit increase (at child age 10 years)	1.05 (1.03 to 1.07)	1.05 (1.03 to 1.07)	1.04 (1.02 to 1.06)
Fathers’ socio-occupational class (child's age 10 years)			
Managerial	Ref	Ref	Ref
Professional	1.48 (1.09 to 2.00)	1.44 (1.06 to 1.95)	1.14 (0.83 to1.57)
Intermediate (skilled and non-skilled)	2.50 (1.88 to 3.33)	2.43 (1.80 to 3.27)	1.55 (1.14 to 2.11)
Routine/manual	3.51 (2.58 to 4.78)	3.56 (2.58 to 4.92)	2.05 (1.47 to 2.87)

*Multivariate model 1 mutually adjusted for all baseline independent variables.

†Multivariate model 2 additionally adjusted for: self-rated health age 42 (excellent; very good; good; fair; poor); participation in vigorous sports age 42; assessment of own weight at age 42 (about right; underweight; overweight; very overweight).

Some differences between univariate and final adjusted models exist; in univariate models girls watched significantly less TV than boys, and children whose fathers were professional workers watched significantly more TV than managerial. However, these associations were no longer apparent in final adjusted models.

### Cross-sectional associations with TV viewing at age 42

Watching TV ≥3 h/day was associated with reporting fair or poor health (fully adjusted OR, 2.10, 95% CI 1.69 to 2.61) in comparison to those reporting excellent health. Those participating in vigorous sports at least once a week were less likely to watch ≥3 h TV per day (OR, 0.75, 0.65 to 0.85); watching ≥3 h TV per day was associated with self-reported overweight/obese (OR 1.60, 1.31 to 1.95).

### Sensitivity analyses

The pattern of results did not change when we replaced fathers’ BMI with mothers’ BMI. Mothers’ BMI was also associated with TV viewing at age 42 (per unit increase in final adjusted model, OR 1.04; 1.03 to 1.06). When we introduced participants highest educational attainment at age 42 into the final model, the association of fathers’ socio-occupational class with TV viewing was attenuated but remained in the model. Compared with managerial, having fathers from professional, intermediate, routine/manual socio-occupational classes was associated with 1.10 (0.79 to 1.52), 1.47 (1.08 to 2.01), 1.96 (1.40 to 2.75) higher odds of greater TV viewing at age 42. Participants’ educational attainment at age 42 was also independently associated with greater TV viewing at age 42; compared with participants with higher education, having only A-levels, GCSEs/O-levels, or no education was respectively associated with 1.62 (1.28 to 2.06), 2.93 (2.44 to 3.53), 3.16 (2.61 to 3.84) higher odds of greater TV viewing.

## Discussion

This study found that children who reported ‘often’ watching TV at baseline were significantly more likely to watch >3 h/days at 32-year follow-up, as were those who had a father in the lowest occupational class compared with managerial. Interestingly, BMI at age 10 was inversely associated with watching high TV in adulthood although the converse was observed for fathers BMI.

To the best of our knowledge this is the first study to investigate early life correlates of TV viewing time in middle age adults in a large representative birth cohort. Two childhood correlates were identified at aged 10 years—TV viewing time and BMI—that were associated with high TV viewing time at aged 42 years. Participants who reported often watching TV at baseline were likely to watch a higher amount of TV at follow-up. This supports previous literature that suggests health behaviours may track from childhood to adulthood^11^ and potentially supports the hypothesis that childhood interventions may also be effective interventions to promote healthy behaviours in adulthood. For example, displacing TV viewing time with physical activity during childhood may mean physical activity will track into adulthood and not sitting time. A somewhat surprising finding was the inverse association between BMI at age 10 and high TV viewing at follow-up. Childhood BMI should, however, be interpreted cautiously as BMI differences in normal weight children can be largely due to fat-free mass.^16^ ^17^

Two parental correlates at aged 10 years were associated with higher TV viewing at follow-up: father's BMI and socio-occupational-class. Participants who had a father in the lowest socio-occupational-class at baseline were significantly more likely to watch greater TV at follow-up, and this was independent of participants’ own highest educational attainment. Previous research has consistently shown that social status from birth has a cumulative effect on poor health in adulthood (eg, see: Power and Hertzman,^18^ Poulton *et al*^19^ and Melchior *et al*^20^). This finding, although using a broader exposure, supports previous cross-sectional research that has shown participants from a lower social status background (measured from a range of indicators—see: Stamatakis *et al*^21^) watch significantly more TV, and tend to be more likely to have multiple TVs in their home. This association may be partially explained by the fact that those from a lower SES are more likely to be physically active at work^22^ and may compensate for this activity with higher levels of leisure sedentary behaviour.^21^ The present study has also shown, for the first time, that participants who had a father with a higher BMI were significantly more likely to watch higher TV at 32-year follow-up than those who had a father with a lower BMI. Assuming that fathers’ higher BMI is indicative of lower physical activity levels, their children may be more likely to adopt sedentary behaviours through modelling their father's activity patterns. Indeed past data have suggested that parental participation in physical activity may be a predictor of childhood activity levels.^23^ An analysis of data from 428 children aged 4–5 years found that those whose parents were either obese/overweight had a stronger preference for sedentary activities, and spent more time in sedentary pastimes than those who had parents of normal-weight/lean.^24^ Taken together, the two parental correlates that we have identified suggest that parents’ TV habits may at least partly influence children's TV habits. This has important implications for policy and practice, by suggesting that interventions to reduce passive TV viewing time should target children and their parents. Intervening now in children and their parents may be a successful strategy to reduce passive TV viewing time in our future generations. Potential interventions may target ‘family time’ and aim to displace passive TV viewing with active screen-based alternatives (eg, active computer gaming), or outdoor activities that encourage movement (eg, evening walks). Specifically, interventions focusing on reducing early life inequalities on TV viewing might have the potential to prevent a widening of inequalities in later life.

Limitations include mother-reported TV viewing time and participation in sport, at age 10, a measure that has not been validated. However, previous literature has shown that mother-reported questionnaires on correlates of physical activity, in preschool children, have shown reasonable validity and internal consistency.^25^ The authors are unaware of similar literature in older children. Whereas measures at baseline were mostly based on parental report, data at 32-year follow-up were based on self-report, and inconsistencies between parent and participant may have introduced bias. It is possible that parents and/or participants may have misreported TV viewing time. For example, parents may have underestimated TV viewing time in fear of being judged for bad parenting. Moreover, owing to its passive nature and high prevalence, TV viewing may be difficult to recall accurately. Nevertheless, TV viewing at age 42 in the present study is broadly comparable with data on TV viewing from Health Survey for England (http://www.hscic.gov.uk/catalogue/PUB00430/heal-surv-phys-acti-fitn-eng-2008-rep-v2.pdf), which is a representative sample of the general English population. The data are, however, from a specific birth cohort and associations may not be generalisable to other birth cohorts. Strengths of this study include its large population-based sample of English, Scottish and Welsh adults and its prospective design with a 32-year follow-up. These findings add to the growing body of cross-sectional literature on TV viewing correlates in adults^11^ and further support the implementation of interventions to reduce passive TV viewing time or make TV viewing active rather than predominantly passive to benefit public health.

## Conclusion

Findings from the present analyses suggest that childhood TV viewing time and body weight may be important correlates of adult TV viewing time. Findings also suggest that fathers’ TV habits may determine children's viewing habits, which may have important implications for the direction of future policy and practice. Specifically, findings support the case for early life interventions, particularly on socioeconomic inequalities, as a way of preventing sedentary behaviour in later life.

**What is already known on this subject**

- Prolonged sedentary behaviour is detrimental to adult health; one important component of adult leisure sedentary behaviour is TV viewing. To successfully modify TV viewing patterns, correlates of the behaviour need to be identified. Crucially, what is missing from the current literature is empirical evidence tracking TV viewing time from childhood through to adulthood and identified childhood correlates of adult TV viewing time.

**What this study adds**

- Childhood TV viewing time appears to track into adulthood. Parents’ health behaviours and social status appear to be associated with their children's TV viewing habits at 32-year follow-up. Interventions to reduce passive TV viewing time should target children and their parents.
